# Weight, physical activity and dietary behavior change in young mothers: short term results of the HeLP-her cluster randomized controlled trial

**DOI:** 10.1186/1475-2891-8-17

**Published:** 2009-05-01

**Authors:** Catherine B Lombard, Amanda A Deeks, Kylie Ball, Damien Jolley, Helena J Teede

**Affiliations:** 1 Jean Hailes Foundation Research Unit, School of Public Health and Preventive Medicine, Monash University, Australia; 2 Monash Institute of Health Services Research, School of Public Health and Preventive Medicine, Monash University, Australia; 3 School of Exercise and Nutrition Sciences, Deakin University, Australia

## Abstract

**Background:**

Preventing weight gain rather than treating established obesity is an important economic and public health response to the rapidly increasing rates of obesity worldwide. Treatment of established obesity is complex and costly requiring multiple resources. Preventing weight gain potentially requires fewer resources to reach broad population groups, yet there is little evidence for successful interventions to prevent weight gain in the community. Women with children are an important target group because of high rates of weight gain and the potential to influence the health behaviors in family members.

**Methods:**

The aim of this cluster randomized controlled trial was to evaluate the short term effect of a community-based self-management intervention to prevent weight gain. Two hundred and fifty mothers of young children (mean *age 40 years ± 4.5, BMI 27.9 kg/m*^2 ^*± 5.6*) were recruited from the community in Melbourne, Australia. The intervention group (n = 127) attended four interactive group sessions over 4 months, held in 12 local primary schools in 2006, and was compared to a group (n = 123) receiving a single, non-interactive, health education session. Data collection included self-reported weight (both groups), measured weight (intervention only), self-efficacy, dietary intake and physical activity.

**Results:**

Mean measured weight decreased significantly in the intervention group (-0.78 kg 95% CI; -1.22 to -0.34, p < 0.001). Comparing groups using self-reported weight, both the intervention and comparison groups decreased weight, -0.75 kg (95% CI; -1.57 to 0.07, p = 0.07) and -0.72 kg (95% CI; -1.59 to 0.14 p = 0.10) respectively with no significant difference between groups (-0.03 kg, 95% CI; -1.32 to 1.26, p = 0.95). More women lost or maintained weight in the intervention group. The intervention group tended to have the greatest effect in those who were overweight at baseline and in those who weighed themselves regularly. Intervention women who rarely self-weighed gained weight (+0.07 kg) and regular self-weighers lost weight (-1.66 kg) a difference of -1.73 kg (95% CI; -3.35 to -0.11 p = 0.04). The intervention reported increased physical activity although the difference between groups did not reach significance. Both groups reported replacing high fat foods with low fat alternatives and self-efficacy deteriorated in the comparison group only.

**Conclusion:**

Both a single health education session and interactive behavioral intervention will result in a similar weight loss in the short term, although more participants in the interactive intervention lost or maintained weight. There were small non-significant changes to physical activity and changes to fat intake specifically replacing high fat foods with low fat alternatives such as fruit and vegetables. Self-monitoring appears to enhance weight loss when part of an intervention.

**Trial registration:**

ACTRN12608000110381

## Introduction

Preventing weight gain is an important economic and public health response to the rapidly increasing rates of obesity. In Australia, almost 60% of the population are considered overweight or obese [1]. Treating established obesity is complex, requiring intensive education, counseling, multiple resources and ongoing support with limited efficacy [2].

To date, there are few reports of successful community-wide weight gain prevention interventions, and limited evidence from smaller targeted interventions. A systematic review in 2000, of interventions specifically to prevent weight gain revealed four published reports in adults based on two separate trials, but only one report of a successful outcome [3]. Subsequent reports on interventions to prevent weight gain produced conflicting results [4,5]. A more recent review reported interventions focusing on diet and physical activity to prevent weight gain found only 9 studies and of these, four positive studies [6]. Lower intensity, local, community-based interventions have the potential to support lifestyle change but need to be tested in diverse groups.

Women are an important target group as they are at high risk of ongoing weight gain. Longitudinal population studies report adult women are increasing weight at a mean rate of 600 g/year [7,8]. Small increases in body mass index (BMI) even within the normal weight range have been associated with increased risk of chronic disease such as diabetes [9]. Women with young children are at particular risk, yet studies rarely target this group. These women also make many of the daily food and activity decisions for families, influencing their children and partner's eating and physical activity levels [10]. Targeting women with children to prevent weight gain has the potential to achieve significant health benefits for themselves and their families.

Possible success factors for behavioral change include social support, goal setting and self monitoring. Self monitoring has been associated with improved maintenance after substantial weight loss in obese individuals [11] but has not been widely tested for the prevention of weight gain in healthy weight individuals. Self-efficacy is a possible predictor of behavior change and weight loss [12,13]. Interventions that enhance self-efficacy beliefs therefore have the potential to improve outcomes. Outcomes may also be enhanced through more effective delivery formats such as making programs tailored and interactive with frequent messages on diet and physical activity [14]. Interventions that are low intensity, low cost, address barriers to participation and achieve sustainable behavior change are urgently needed.

The primary objective of this study was to examine the short term effect of a longer term intervention, the Healthy Lifestyle Program (HeLP-her) which was specifically designed to prevent weight gain in women with young children living in the community. Here we report on the weight, weight related behaviors and self efficacy in participants following phase 1 of the intervention.

## Methods

### Participants

The target population were generally healthy, community-based mothers of at least one school-aged child (5–13 years) attending a primary school. Subjects were excluded if they were taking weight control medications, pregnant or became pregnant during the study, were breastfeeding infants under 6 months of age, or who wished to gain weight. To be inclusive of all community members, BMI was not used as an inclusion or exclusion criterion.

### Recruiting

Twelve schools were recruited, paired with a school of equal size and within each pair, one school was randomly allocated to intervention (n = 6), the other to control (n = 6) using computer generated numbers. All mothers of children who attended these primary schools were invited by letter to participate and register interest by return mail, phone, email or fax. The study sampling and intervention were designed to target clusters of mothers associated with particular schools to reduce possible contamination between participants. The research teams randomized the schools and because they delivered the program were aware of the allocation of schools to intervention or control. Scoring and data entry was performed by team members blind to the allocation of participants.

Two hundred and fifty women were recruited progressively from April 2006 to August 2006. These women were given information packs containing an explanation of the study, questionnaires, completed a written consent and were randomized to intervention (n = 127) or control (n = 123) according to their school association.

Prior to attending group sessions, women were asked by survey to estimate their current weight. Participants were weighed and measured at the first group session and received a request for a fasting blood sample. Participants were given results of baseline measurements including blood tests after recruitment so as not to influence participation.

### Intervention phase 1

The intervention content was based on the social cognitive theory, specifically, goal setting, self monitoring, social support, problem solving and relapse prevention training offering multiple avenues to behavior change [15]. In phase 1, participants attended four sessions; three one hour interactive group sessions in the first month, plus one review session at four months delivered by an experienced Dietitian. Content included evidence based messages with clear goals on diet, (e.g. eat 2 serves fruit and 5 serves vegetables each day), physical activity (e.g. aim for 8,000–10,000 steps per day) and behavior change (e.g. monitor yourself regularly). Written handouts were provided. In the following sessions outcome expectancies were discussed to clarify the intervention aim to prevent weight gain, not to promote weight loss. Regular daily or weekly self monitoring of weight was strongly encouraged. Women were encouraged to enter voluntary school based walking groups or to walk with friends for social support. A pedometer was provided as a voluntary self monitoring tool and women were given instructions to aim for at least 8,000–10 000 steps per day. A final visit at four months reinforced lifestyle and behavioral messages and collected data in the intervention group. All sessions were held in groups, ranging in number from 10–30 participants, at the local primary school. Ongoing support was provided through one contact per month via text messages, phone calls or email encouraging and reminding participants of the diet, physical activity and behavioral strategies discussed in the group sessions.

### Comparison group

The comparison group attended a single thirty minute, non-interactive health education group lecture based on the Australian Dietary Guidelines and the Australian Physical Activity Guidelines. They received readily available pamphlets based on these Guidelines [16,17]. They received a pedometer to use as they wished over the year, but no daily step goal. They received no further support, but completed a brief mailed questionnaire at 4 months, and returned for final data collection at 12 months.

### Study Measures

#### Anthropometric measures

Self reported weight data was collected in both groups at baseline and at 4 months. Measured weight using a calibrated electronic scale (Tanita model BWB-800 digital scale) was collected in both groups at baseline and the intervention only at 4 months. We did not weigh the control group at 4 months to avoid any potential intervention effect of weighing, as the long term study is ongoing.

#### Physical Activity

The International Physical Activity Questionnaire short version (IPAQ) has been validated and was used to measure usual weekly physical activity [18,19]. Physical activity was expressed as a continuous variable; MET-minutes per week (MET-mins = MET level × minutes per day × days per week) where 1 MET is equivalent to resting energy expenditure and according to IPAQ categories, walking, moderate and vigorous

#### Diet

Fat intake was measured using the validated Fat Behavior Questionnaire (FBQ), a reliable and responsive measure of dietary fat intake [20]. The FBQ has been used in dietary studies and reported correlations with fat intake for fat related subscales using food diaries and food frequency questionnaires ranging from 0.60–0.77. FBQ is measured on a Likert scale; a mean score is generated overall, along with individual sub-factors. Low scores indicate low fat intake and high scores indicate high fat intake.

#### Self monitoring

To assess self monitoring we asked both groups at baseline, and again at 16 weeks how often they weighed themselves; daily, weekly, monthly, occasionally or never. Those who weighed daily or weekly were considered regular self weighers and those who weighed themselves monthly, occasionally or never, were categorized as non-regular self weighers.

#### Self efficacy

The Eating and Exercise Confidence Scale developed by Sallis was adapted to measure self efficacy [21]. The reported reliability for the domains of physical activity and eating are 0.68 and 0.43–0.68 respectively and internal consistency was 0.83–0.85 and 0.85–0.93 respectively. Wording in the eating scale was modified to match Australian foods. Questions related to salt intake were omitted as they were irrelevant to this intervention. An additional question was included, 'how confident are you that you can control your weight if you wished?' to measure self efficacy for controlling weight. Scoring was based on a likert scale, a mean of correctly completed questions generated for each domain.

Ethics approval for this study was obtained through the Southern Health Human Research Ethics Committee.

### Statistical Methods

To account for the clustered design we used an intra-cluster correlation of 0.02 and an estimated cluster size of 30 to determine sample size. We calculated that 110 participants, 5–6 clusters, were therefore required for each group at 90% power to detect at least a 600 g difference in weight between intervention and controls, the mean annual weight gain in women.

Data were analyzed at the participant level. First sample means, standard deviations (SD), and proportions were calculated for relevant demographic characteristics of the population and differences assessed using chi^2^. The main outcome was a difference in weight change between groups at 16 weeks. Firstly, self reported weight was analyzed using linear regression, where individual differences in weight between baseline and week 16 were derived, the difference regressed on group assignment, adjusting for the clustering effect of school and thereby generating appropriate confidence intervals. Secondly, a difference in measured weight between baseline and week 16 in the intervention group only was calculated and analyzed using regression as described above. Within group changes from baseline to 16 weeks in all variables, were analyzed using t-tests. Differences between groups in all variables were measured using linear regression, after first generating a difference in the score from baseline, and adjusting for clustering as described above. Analysis was based on the women who returned for measurement and reported their weight via mailed survey. Intention-to treat analysis will be performed on the final data collected at 12 months.

Self monitoring was analyzed by dividing the participants into two categories, regular self-weighers (daily or weekly) and non-regular self-weighers (occasional or never). We used linear regression to estimate the association between regular self-weighing and weight change, using the difference in self reported weight from baseline to four months as the dependent variable and the self weighing categories as independent variables. All data were analyzed using Stata 9 (Statacorp, Texas, USA) statistical software program.

## Results

**Figure 1:**
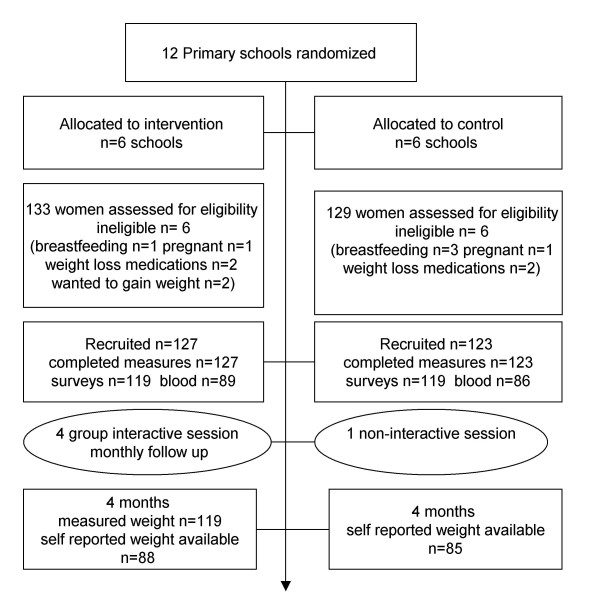
**1 Flow chart of subject enrolment, random assignment and completion intervention delivery**.

Figure 1, shows the flow of participants through the study. Three hundred women responded to our invitation (11%) and 250 participants were recruited. Differences in demographic characteristics at baseline between intervention and comparison groups were small (Table 1). One hundred and seventy three returned the 4 month questionnaire.

**Table 1:** Baseline characteristics of the HeLP-her intervention by group assignment

Variable	Intervention group (n = 127)	Control group (n = 123)	P value^a^
Age at baseline, mean (SD), yr	40.6 (4.8)	40.3 (4.8)	0.62
Height Mean (SD), cm	163.1 (6.0)	162.9 (5.6)	0.78
Weight, kg Weight Range, kg	73.2 (13.8) 46.3 to 115.6	74.6 (16.1) 44.6 to 129.6	0.45
BMI Mean,(SD)^b ^kg/m^2^	27.5 (5.1)	28.1(5.8)	0.40
Hip, cm	106.6 (11.0)	108.1(11.8)	0.29
No. of children, mean, (SD)	2.3 (0.7)	2.4 (0.8)	0.23
Waist circumference mean, (SD), cm	94.8 (12.6)	96.8 (14.6)	0.24
Energy intake (kJ/day)^d^	6648 (2204)	6830 (2361)	0.55
Energy expenditure Met-mins/week^e^	1504 (1657)	1653 (452)	0.52
Highest Education			
Up to year 10 n (%)	25 (21.0)	36 (30.3)	
Year 12	25 (21.0)	26 (21.9)	0.31
Trade or certificate	33 (27.7)	24 (20.2)	
University or higher	36 (30.3)	33 (27.7)	
Income ($ AUD)^c^			
< 40,000	22 (18.8)	27 (22.8)	
40–60,000	26 (22.2)	18 (15.2)	0.08
60–80,000	20 (17.0)	26 (22.0)	
> 80,000	31 (26.4)	33 (27.9)	
No answer	18 (15.3)	14 (11.8)	
Employment (%)			
Not working	46 (40.3)	49 (41.1)	
Part time	61 (53.5)	63 (52.9)	0.99
Full time	7 (6.1)	7 (5.8)	

Abbreviations, MET-mins/week = MET level × minutes per day × days per week, where 1 MET is equivalent to resting energy expenditure (MET level, walking, 3.3, moderate 4.0 and vigorous 8.0)

^a ^P values from 2-sample t tests for continuous variables or from chi2 tests for categorical variables

^b ^Body mass index, measured as weight in kilograms divided by height in meters squared

^c ^Numbers may vary due to missing values or incorrectly completed surveys as participants were given a 'prefer not to answer' option for income.

^d ^n = 110 intervention and n = 106 controls correctly completed food frequency questionnaires

^e ^n = 89 intervention and n = 89 controls correctly completed IPAQ activity questionnaires

### Weight Change

**Figure 2:**
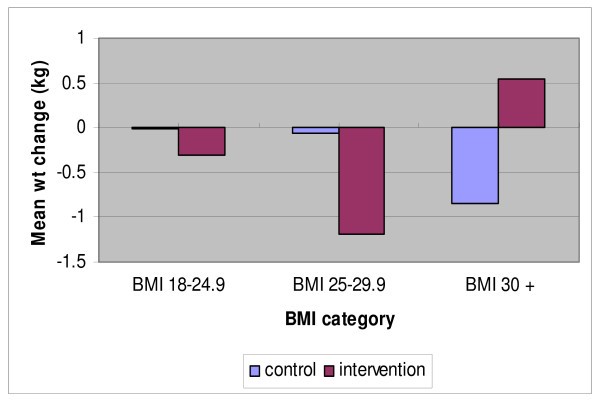
**Mean weight change according to BMI category**. Control group BMI < 25 n = 24, BMI 25–29.9, n = 38, BMI ≥ 30 n = 20. Intervention group BMI < 25 n = 37, BMI 25–29.9 n = 28, BMI ≥ 30 n = 21.

**Figure 3:**
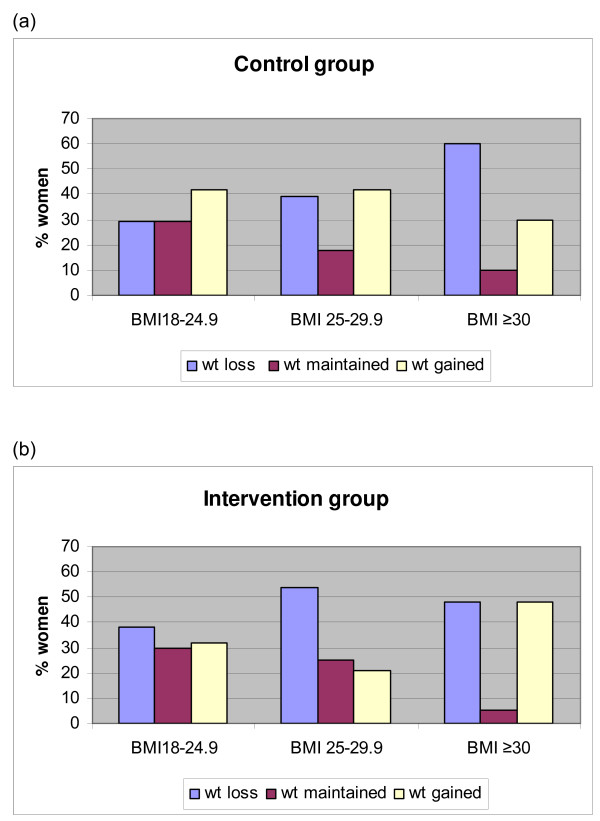
**Percentage of participants who lost, maintained or gained weight**. (a) Control group BMI < 25 n = 24, BMI 25–29.9, n = 38, BMI ≥ 30 n = 20. (b) Intervention group BMI < 25 n = 37, BMI 25–29.9 n = 28, BMI ≥ 30 n = 21.

Using self-reported weight, both control and intervention groups reported a similar but non-significant mean weight loss at 16 weeks, -0.72 kg (95% CI:-1.59 to 0.14 p = 0.10) and -0.75 kg (95% CI:-1.57 to 0.07, p = 0.07) respectively. However, based on the measured weight, the intervention group participants significantly reduced weight by -0.78 kg (95% CI: -1.22 to -0.34, p < 0.001) (see additional file 1). There were differences between groups in the distribution of weight loss after stratification by BMI category, although this did not reach significance (figure 2). We then further stratified women into those who lost weight (≥ 1 kg), maintained weight within 1 kilogram of baseline (+/- 0.9 kg) to account for day to day variation in weight, or gained weight (≥ 1 kg). More women in the intervention group lost or maintained weight compared to the comparison group, particularly if they were overweight at baseline (figure 3). Again, the difference between groups did not reach significance. The obese category reported some large increases or decreases in weight over this short period (figure 3).

### Weight monitoring

Intervention participants who regularly weighed themselves reported mean self-reported weight loss at four months (-1.66 kg), significantly different to those who did not weigh themselves (+ 0.07 kg), a difference of -1.73 kg, (95% CI: -3.35 to -0.11, P = 0.04). This was different to the control group where although regular self-weighing was associated with weight loss, the difference in reported weight change between the regular weighers and non-weighers was not significant (-1.41 kg (95% CI: -3.18 to 0.27, P = 0.1).

### Diet and physical activity behaviors

The fat-behavior questionnaire measured 5 sub-factors of fat intake behavior where low scores are associated with low fat intake. The difference between intervention and control groups in dietary fat behavior at four months is reported (see additional file 1).

Both groups increased physical activity; the intervention reported greater increases, although the difference between groups did not reach significance (see additional file 1).

### Self Efficacy

Self-efficacy (SE) beliefs were measured in three domains on a Likert scale as confidence to control weight, improve diet and participation in physical activity. There was a decrease in all three self-efficacy domains in the comparison group, with a significant decrease in both diet and physical activity domains. Self-efficacy in the physical activity domain decreased in the intervention group although no differences between groups were detected.

## Discussion

Prevention of weight gain is an important step in reducing the impact of obesity on population health, yet there are few reports of successful interventions to date. Here we have reported on a low intensity intervention specifically to reverse the current trend of weight gain seen in Australian women.

Self-reported weight is commonly used in interventions due to the convenience and low cost. While it is considered to under report actual weight, it has been suggested self-report may accurately reflect changes in weight [22]. We did not weigh the control group at 4 months to avoid any unplanned intervention effect of weighing as the longer term study was still in progress. While we cannot be sure the self-reported weight changes described are not due to under-reporting, both groups were weighed at baseline and this may have contributed to more accurate self-reporting at 4 months.

It appears a similar small weight loss has occurred in both the comparison group and the intervention group. The weight change itself is of limited clinical importance because of the short term nature of the study, but it does point to interesting short term behavioral change in women with young children who are difficult to engage in health interventions. Previous reports including our own suggest the majority of women are dissatisfied with their weight even those within a healthy BMI range, and frequently use weight loss behaviors [23,24]. It is interesting then that even a single education session in the control group resulted in small weight loss in the short term. The program may have influenced early motivational changes in women as described in the Health Education Model, or influenced women at the contemplation and action stage as described in the Trans Theoretical Model. As participation was voluntary there is potential for a more motivated group to be recruited in both groups and this may partly explain the weight loss. However the women were invited to attend a lifestyle program, not a weight loss program and we anticipated we might recruit those who were already active, consuming a healthy diet and maintaining their weight. However the recruited participants included those who were overweight and obese as well as normal weight and the proportion who were inactive was similar to population levels as we have reported previously[25]. So whilst some women may have attended with the intention of losing weight, we do not believe the majority of women volunteered with this intention. If in fact we did recruit a sample that was already engaging in healthy lifestyle behaviors we would expect to see an attenuated effect among the respondents, and hence the effect size is an under-estimate of the effect which would be seen in all women. This suggests that being weighed and some simple general guidelines provided in a community setting may be sufficient to assist short term behavior change leading to better weight control in some women, at least in the short term. The greater weight loss reported in overweight and obese women suggests that even a single education session will encourage significant weight loss in some, but not all, overweight and obese women. Previous studies suggest this is not likely to be sustained long term. It is possible some women respond well to community interventions and it may be important to determine how these women can be identified to improve outcomes. Of concern is the large number of women who reported weight gain in each BMI category even in this short period.

The intervention group not only achieved weight loss, but there was a trend toward small, but potentially important changes to health related behaviors such as physical activity. Accurately measuring small changes to dietary intake and physical activity is difficult due to the lack of sensitive tools. We were unable to determine a difference between groups in fat intake behaviors in the short term. Both groups reported replacing high fat foods and snacks with lower fat alternatives such as fruit and vegetables, which potentially reduces the overall energy density of the diet and possibly contributed to the small energy deficit and weight loss. Some of the lower fat behaviors such as avoiding frying food and using low fat meats are well known messages throughout the community. Some women may have already been practicing these behaviors resulting in little further change attributed to the intervention. However, the small improvements in physical activity and diet and the associated changes in energy balance are consistent with that expected to prevent population weight gain long term, estimated to be as little as 60 kJ per day [26]. The weight change reported here is important if sustained long term in populations [27]. The longer term results of this study will help determine the clinical importance and sustainability of the small energy deficit and weight change.

Self-weighing appears to facilitate weight loss when included as part of a behavior change intervention. There is only one report of using self-weighing in interventions specifically to prevent weight gain, although there are reports of the successful use of weight monitoring in obesity treatment and the subsequent prevention of weight re-gain [28]. Our results confirm those seen in the 'Pound of Prevention' a large community prevention intervention, where at least weekly weighing was associated with weight loss, although they did not show a difference between intervention and control. The study showed no detrimental effect of self weighing [14]. Simple methods of monitoring progress such as self-weighing may be a viable population recommendation although potential detrimental effects would need to be fully evaluated first.

Self-efficacy has been reported to be a predictor of successful behavior change. In this study, we were not able to show a correlation between self-efficacy and weight change despite positive correlations in other studies [29]. Self-efficacy declined over this short period, particularly in the control group. Intervention components such as outcome expectancy discussions, social reinforcement and modeling may have helped maintain self-efficacy in the intervention participants. The decline in self-efficacy for physical activity cannot be explained easily, although Bandura theorized that past successful experience is a powerful influence on self-efficacy and is supported by a study by Hofstetter [30]. Declining self-efficacy for physical activity in women with children has also been demonstrated previously [31]. This group may therefore need additional support to maintain self-efficacy for physical activity behaviors. Possibly, failure to employ the planned physical activity may have led to a decline in confidence to participate in future activity. This is explained partly through research on mediators of self-efficacy [32].

This study is important as it is the first to target mothers of young children specifically for the prevention of weight gain. This intervention recruited a representative sample of community dwelling women with children in Australia which along with other baseline characteristics has been reported previously [25]. Overall approximately 11% of invited participants responded. In the context of population reach the school setting gave access to all available women with children, unselected in terms of health risks. Reaching and changing the behavior in 11% of women with children translates into a substantial health promotion intervention

We were not able to show a significant difference between the groups in weight change and some specific lifestyle behaviors. The inability to detect these differences may reflect the inadequacy of the available self-report questionnaires to detect small changes. We cannot eliminate the possibility that self-reported improvements in diet and physical activity may also be the result of positive response bias. It is also possible seasonal variation contributed to the weight change although this was limited by having a rolling period of data collection. The study was powered to detect a 600 g difference between groups in the long term, and not adequately powered to detect the small difference observed here at four months. The intervention aimed for small consistent changes to lifestyle and this small energy deficit is difficult to measure with available tools. Despite this, the weight change and the behavior change seen in the intervention group is encouraging for the longer term success of this intervention.

## Conclusion

Both, a low-intensity, interactive, lifestyle intervention as well as a single group health education session prevented weight gain and resulted in small weight loss in the short term in community based women with children. More intervention women reported weight loss or maintenance than in the comparison group. There were small changes to diet and physical activity behaviors in both groups, specifically replacing high fat foods with low fat alternatives such as fruit and vegetables. Regular monitoring of weight appeared to enhance weight loss associated with the intervention group. Further investigation will establish the sustainability and long term effectiveness of this intervention on the prevention of weight gain

## Authors' contributions

CL HT DJ designed the study. CL DJ analysed the results. KB AD contributed to the psychological and behavioural aspects of the design and intervention components and contributed to the interpretation of results and drafts of this paper. CL was responsible for design, recruiting, delivering the intervention and arranging and supervising the collection of data, analysing the data and writing the manuscript. HT provided intellectual input into all aspects of the design, data collection, analysis and reporting. All authors read and approved the final manuscript.

## Competing interests

The authors declare that they have no competing interests.

## Supplementary Material

Additional file 1 **Change from baseline to 4 months in weight related behaviors (mean, 95% CI) according treatment groups, and differences between groups at 4 months (mean difference, 95% CI)**. Describes changes to weight, fat intake behaviors, self efficacy and physical activity according to treatment group. Click here for file
