# Supplementary material for: Weight, physical activity and dietary behavior change in young mothers: short term results of the HeLP-her cluster randomized controlled trial
Source: Nutr J. 2009 May 1;8:17. doi: 10.1186/1475-2891-8-17 (PMC2683875; doi:10.1186/1475-2891-8-17)
Supplement: Additional file 1 — Change from baseline to 4 months in weight related behaviors (mean, 95% CI) according treatment groups, and differences between groups at 4 months (mean difference, 95% CI). Describes changes to weight, fat intake behaviors, self efficacy and physical activity according to treatment group. [file 1475-2891-8-17-S1.doc]

Change from baseline to 4 months in weight related behaviors (mean, 95% CI) according treatment groups, and differences between groups at 4 months (mean difference, 95% CI).

|  | Control | | | Intervention | | |  |  |
| --- | --- | --- | --- | --- | --- | --- | --- | --- |
|  | Baseline | 4 months | Change over time ^1^  (95% CI) | Baseline | 4 months | Change over time ^1^  (95% CI) | Difference in scores between groups adjusted ^2^ (95% CI) | P values |
| **Weight (kg)**  **Self reported**  **Weighed** | 73.77  74.61 | 73.05  N/A | n=85 ^3^  -0.72 ( -1.59 to 0.14) p=0.10 | 71.98  73.38 | 70.23  72.59 | n=88  -0.75 (-1.57 to 0.07) p= 0.07  n=119  -0.78 ( -1.22 to -0.34) *** | -0.03 (-1.32 to 1.26) | 0.95 |
| **Fat behaviors ^4^**  Modify meat  Avoid frying  Avoid high fat foods  Substitute with low fat  Replace high fat foods | 1.48  1.92  1.74  1.78  1.96 | 1.45  1.90  2.09  1.91  1.89 | n=85  -0.02 (-0.11 to 0.05)  -0.02 (-0.14 to 0.10)  +0.35 (0.22 to 0.48)***  +0.13 (0.04 to 0.22)**  -0.07 (-0.14 to -0.07) * | 1.48  1.84  1.72  1.76  1.90 | 1.41  1.79  2.08  1.90  1.76 | n=100  -0.07 (-0.19 to 0.05)  -0.05 (-0.18 to 0.07)  +0.36 (0.20 to 0.51)***  +0.14 (0.05 to 0.23)***  -0.14 (-0.21 to -0.08) ** | -0.04 (-0.25 to 0.17)  -0.03 (-0.27to 0.21)  0.01 (-0.22 to 0.24)  0.01(-0.08 to 0.11)  0.07 (-0.22 to 0.07) | 0.68  0.76  0.93  0.80  0.28 |
| **Self-efficacy**  Weight  Diet  Physical activity | 2.86  3.27  2.74 | 2.81  3.09  2.44 | n=88  -0.04 (-23 to 0.11)  -0.18 (-0.26 to -0.08)*** -0.29 (- 0.42 to-0.16)*** | 3.11  3.27  2.86 | 3.09  3.14  2.49 | n=100  -0.02 (-20 to 0.16)  -0.12 (-0.25 to 0.00)  -0.37 (-0.51to-0.24)*** | 0.26 (-0.20 to 0.16 )  0.05 (-0.16 to 0.26)  -0.08 (-0.31 to 0.41) | 0.82  0.61  0.44 |
| **Physical Activity**  Walking MET-mins  Moderate MET-mins  Vigorous MET-mins | 767  262  344 | 826  391  530 | n=73  +58 (-25 to 372)  +129 (-60 to 318)  +186 (-125 to 496) | 858  346  367 | 952  543  600 | n=71  +94 (-256 to 445)  +197 ( -119 to 513)  +233 (-19 to 485) | 35 (-315 to 416)  67 (-389 to 525)  46 (-412 to 506) | 0.84  0.75  0.82 |

( ) = 95% confidence interval

^1^ paired t-test

^2^ difference in change scores between groups (intervention –control) using linear regression adjusted for baseline values and accounting for clustering of schools

^3^ n values represent the number who returned accurately completed surveys with self reported weight both at baseline and 4 months

^4^  fat behavior scores, lower score = lower fat intake, higher scores =higher fat intake

* p ≤ 0.05 ** p ≤ 0.01 *** p ≤ 0.001
